# Bridge the Gap: Reducing Inequity in Hospital Readmissions for African American Patients with Heart Failure Through Quality Improvement Initiatives

**DOI:** 10.1089/heq.2020.0082

**Published:** 2021-01-25

**Authors:** Carolina Ornelas, Raj P. Fadadu, Morrise A. Richardson, Omonivie H. Agboghidi, Jonathan D. Davis

**Affiliations:** ^1^ School of Public Health, University of California, Berkeley, Berkeley, California, USA.; ^2^ School of Medicine, University of California, San Francisco, San Francisco, California, USA.; ^3^ Department of Cardiology, Zuckerberg San Francisco General Hospital, San Francisco, California, USA.

**Keywords:** health equity, heart failure, quality improvement

## Abstract

**Purpose:** Heart failure (HF) disproportionately impacts African Americans. We evaluated existing quality improvement (QI) initiatives and patient and provider perceptions of barriers to HF care to develop equity-centered QI recommendations.

**Methods:** We performed a literature review, interviewed providers and patients (*N*=11), and conducted a root cause analysis at a safety net hospital in San Francisco, California.

**Results:** We have identified four elements to foster a more equitable HF care model: screening for social determinants of health, technological innovation, optimization of space, and implicit bias training.

**Conclusion:** QI initiatives for HF should integrate health equity elements in their design and implementation.

## Introduction

Heart failure (HF) affects more than 6 million people in the United States and has a prevalence expected to increase by 46% from 2012 to 2030.^1,2^ Compared with the general population, African Americans (AAs) are more likely to develop HF at a younger age, experience HF-related hospitalization, and die from HF.^1,3,4^ This highlights a significant health inequity among HF patients related to race and racism. The coronavirus disease 2019 (COVID-19) pandemic further complicates access to care and intensifies factors contributing to health disparities, potentially exacerbating the disproportionate mortality rate among AAs with HF.^5^ The pandemic catalyzes the need to more broadly analyze health care delivery in the context of social determinants of health (SDoH) to inform and sustain quality improvement (QI), which can inform solutions that mitigate health inequities.

Zuckerberg San Francisco General (ZSFG) is a safety net hospital in California serving a patient population with a high prevalence of HF, a diagnosis carried by 40% of all patients in the cardiology clinic.^6^ To improve patient health outcomes, ZSFG launched a few QI projects to build a multidisciplinary approach to HF. QI is an approach used for the continuous and systematic improvement of health and health care systems. Although these efforts lowered hospital readmission rates among all patients with HF, AA men continued to have the highest rates.^6^

To better address this inequity, the ZSFG Heart Failure Clinic partnered with graduate students at the University of California, Berkeley School of Public Health. Their joint QI project consisted of patient and provider interviews and a literature review to conduct a root cause analysis and develop action items for closing the equity gap.

## Methods

A mixed methods approach was implemented in this study to identify potential underlying causes of health inequity in hospital readmission rates at ZSFG for AA patients with HF. Data collected from informational interviews and the literature search were analyzed using QI tools to determine root causes and develop recommendations. Publication of this study's findings is exempt from Institutional Review Board review.

Interviews using semistructured guides were performed with patients diagnosed with HF who were admitted to the hospital and providers who played a role in the management of HF and/or QI at ZSFG. Verbal consent from all participants was obtained and no identifying information was collected. Thematic analysis of interviews was conducted to assess key themes among and between patients and providers.

The literature review was performed using combinations of keywords, including “heart failure,” “disparities,” “inequities,” “race,” and “quality improvement,” on PubMed and Web of Science. Studies were excluded if the content was out of scope and full-text versions were not available.

We synthesized data collected from interviews and the literature search while performing a root cause analysis and developing recommendations for health equity-centered QI initiatives. The results are displayed using a fishbone diagram.

## Results

Semistructured interviews were performed with seven patients admitted with HF (age: 45–70 years; gender: six males and one female; race/ethnicity: four AAs, two Latinos, and one Caucasian) and four health care providers and administrators (cardiology and noncardiology staff). Thematic analysis of patient interviews demonstrated that SDoH impact access to HF care and patient engagement, including medication adherence. Mutual respect between patients and providers, or lack thereof, including discriminatory experiences, influenced patients' perceived quality of care and trust of the health care system. Analysis of provider interviews revealed that communication with primary care staff, standardization of HF management protocols, optimization of technology, and social work coordination are essential components of equity-focused QI efforts for HF management.

**FIG. 1.**
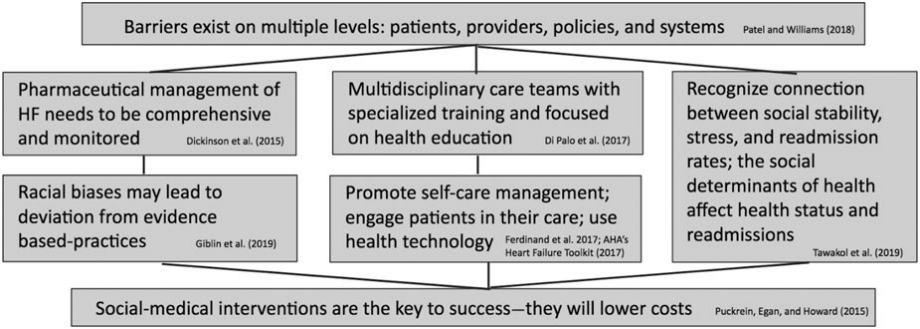
Connections between barriers to accessing care for patients with heart failure that should inform equity-centered quality improvement.

The literature review yielded 55 studies, and after screening studies for relevance, 21 full-text articles/reports were reviewed. The findings from the review bolster many of the themes identified from the analysis of patient and provider interviews: addressing implicit racial bias through provider training, improving patient engagement with technology, and implementing social-medical interventions addressing SDoH.^1,7–13^ Figure 1 demonstrates the connections among the primary identified themes across studies.

**FIG. 2.**
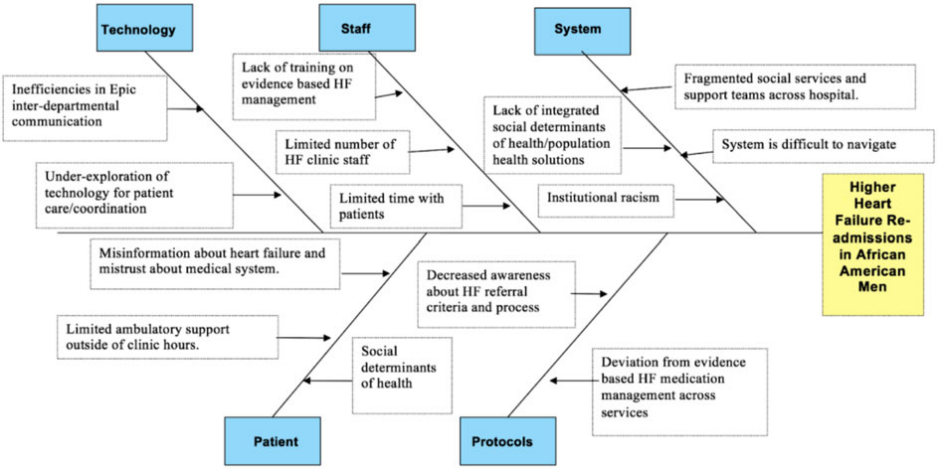
Fishbone diagram highlighting contributing factors to higher readmission rates for African American men who have heart failure.

The root cause analysis integrated information from the interviews and literature search, and the results are presented in a fishbone diagram (Fig. 2). Several root causes were identified that involve multiple stakeholders: patients (misinformation and limited outpatient support), staff (lack of evidenced-based and implicit bias training), protocols (lack of standardization), systems (fragmented social and medical services), and technology (unexplored potential for patient care coordination and follow-up).

## Discussion

Previous studies have examined QI work to improve health outcomes for patients with HF.^9–11^ This study demonstrates the fundamental importance of institutionalizing equity frameworks in QI work, which can be used to address medical and psychosocial barriers to reduce the inequities in readmission rates for AA patients with HF at ZSFG. Aligning patient experiences, providers' perceived gaps in care delivery, and tailored QI approaches in the literature will guide the cultivation of necessary structural and organizational changes to improve health equity for patients with HF. This study's findings of experiences at ZSFG may reflect the broader themes of socioeconomically diverse populations at safety net hospitals that are not comprehensively examined in current literature.

Four key elements are identified to improve the care delivery model for patients with HF. First, there needs to be a robust universal screening for SDoH to connect individuals with support services, such as case management, to promote health and prevent readmissions. For example, dietary changes in HF are important but difficult for our patients who live in communities with disproportionately higher prevalence of unhealthy food options. Without such standardized screening, gaps in support services emerged, impacting patients' ability to take their medicines, attend appointments, and engage in health promoting behavior. Even though clinic appointments are limited in time, we found that staff supported SDoH screening because this information would aid providers in tailoring unique care plans. Fortunately, ZSFG exists in a county with many social support services, but the infrastructure to identify and connect patients with HF must be enhanced.

Second, technology must be better integrated to assist with screening and streamlining care. Staff described the importance of implementing workflows into the electronic health record (EHR) to seamlessly connect patients with HF with case managers and screen them for social needs. Automaticity would limit the technological burdens for clinicians, possibly increasing general buy-in for important SDoH screenings. Digital tools, such as the protocol for responding to and assessing patients' assets, risks, and experiences, which gathers SDoH information, and Aunt Bertha, which connects patients with need-based social services, already exist within major EHRs and should be more readily integrated especially at safety net hospitals.^14,15^

Third, the physical space where patient care is delivered needs to be optimized. The ZSFG Cardiology Clinic is in proximity to primary care clinics, which interviewees noted as a strength because it fostered sharing of resources and staff such as social work and Addiction Medicine. An equitable hospital design must consider how to maximize and link resources within the hospital, including colocation of services, to facilitate multidisciplinary collaborations and more smoothly and rapidly link patients with these essential services.

Fourth, there needs to be comprehensive implicit bias training for medical providers. Patients with HF shared experiences of racism and discrimination in the health care system during their lifetime. For patients to feel safe and welcome, ZSFG must offer robust training on implicit biases at all provider levels, increase the number of health care workers of color, and implement culturally sensitive health education for patients.

COVID-19 magnifies the importance of health equity. COVID-19 is disproportionately impacting AA patients through the similar SDoH and structural inequity contributing to our higher readmission rates for HF patients.^5,16^ This study emphasizes how the existing health care system is not equipped to address racial inequities. However, equity-centered QI can provide opportunities to address social barriers when patients with HF interface with the health care system.

This study has certain limitations. Although there is a limited sample of people who were interviewed, we sought to sample from a range of patient care settings. Further research should be conducted with a larger set of patient and provider interviews. Although all interviews were conducted at one safety net hospital in one city, it is emblematic of a diverse urban underserved environment. The findings should be contextualized to the study's specific hospital and geographic setting. Finally, this study focused on inequities related to patients with HF, so further research is needed to determine recommendations for equity-centered QI work to improve the health of patients with other diseases.

## Conclusion

The need for equity-focused QI to address SDoH for the treatment of HF is crucial. Traditional QI approaches may reduce overall health disparities, but inequity will persist. This study's synthesis of first-hand perspectives from patients and providers, in the context of a literature review, shows that equity-focused QI must acknowledge and address the unique root causes that impact underserved populations, such as AA patients with HF. The findings and recommendations are a first step in shifting our current paradigm through integrating SDoH, optimizing technology and space, and addressing implicit racism within medicine to build innovative care delivery systems that address inequities in our safety net hospitals.

## Abbreviations Used

AAs: African Americans
COVID-19: coronavirus disease 2019
EHR: electronic health record
HF: heart failure
QI: quality improvement
SDoH: social determinants of health
ZSFG: Zuckerberg San Francisco General
